# KMT2A-rearranged B-lymphoblastic lymphomas are skewed towards a more mature developmental stage

**DOI:** 10.1038/s41375-026-02943-0

**Published:** 2026-03-23

**Authors:** Ingram Iaccarino, Alina M. Hartmann, Fatih Yalcin, Mayukh Mondal, Cecilia Bozzetti, Nadine Wolgast, Jan L. C. Loeffen, Judith M. Boer, Monika Brüggemann, Claudia D. Baldus, Gunnar Cario, Wolfram Klapper

**Affiliations:** 1 Department of Pathology, Hematopathology Section and Lymph Node Registry, University of Kiel, Kiel, Germany; 2 Clinical Research Unit CATCH ALL (KFO 5010) funded by the Deutsche Forschungsgemeinschaft (DFG, German Research Foundation), Kiel, Germany; 3 University Cancer Center Schleswig-Holstein (UCCSH), University Medical Center Schleswig-Holstein, Kiel, Germany; 4 Medical Department II, Hematology and Oncology, University Hospital Schleswig-Holstein, Kiel, Germany; 5 School of Biology, Indian Institute of Science Education and Research, Thiruvananthapuram, India; 6 Institute of Clinical Molecular Biology, University of Kiel, Kiel, Germany; 7 Princess Máxima Center for Pediatric Oncology, Utrecht, the Netherlands; 8 Department of Pediatrics I, Pediatric Hematology/Oncology, University Medical Center Schleswig-Holstein, Campus Kiel, Kiel, Germany

**Keywords:** Acute lymphocytic leukaemia, Oncogenesis

## To the Editor

In both B-lymphoblastic leukemia (B-ALL) and Acute Myeloid Leukemia (AML), rearrangements of the *KMT2A* gene (KMT2Ar) are associated with unfavourable outcome. In contrast, in B-lymphoblastic lymphoma (B-LBL), the non-leukemic counterpart of B-ALL, no significant difference in progression-free and overall survival associated with KMT2Ar were observed in our previous study focused on a pediatric patients’ cohort [1]. This discrepancy is remarkable, given that clinical treatment of B-LBL and B-ALL is nearly identical during induction, consolidation and maintenance. B-LBL differs from B-ALL by the presence of tissue manifestation and low ( < 25%) or even absent infiltration of the bone marrow (BM). Given the distinct clinical presentation of KMT2Ar B-LBL compared to B-ALL, as well as the impressive difference in outcome, we asked if B-LBL samples of the KMT2Ar subtype in pediatric patients were characterized by specific molecular features. We also evaluated if these molecular features of B-LBL could be shared with extramedullary (EM) manifestations of KMT2Ar B-ALL. To this end, we identified biopsies of KMT2Ar B-LBL (*n* = 14) and KMT2Ar EM B-ALL (*n* = 6) in the files of the Hematopathology Section of the Kiel University. Tissue biopsies of B-LBL and EM B-ALL patients (*n* = 32) lacking KMT2Ar served as a control. Patients’ characteristics are listed in Table S1. The clinical and molecular features of 27 of these samples (six KMT2Ar and 21 belonging to other subtypes) were already part of our previous publication [2].

**Fig. 1:**
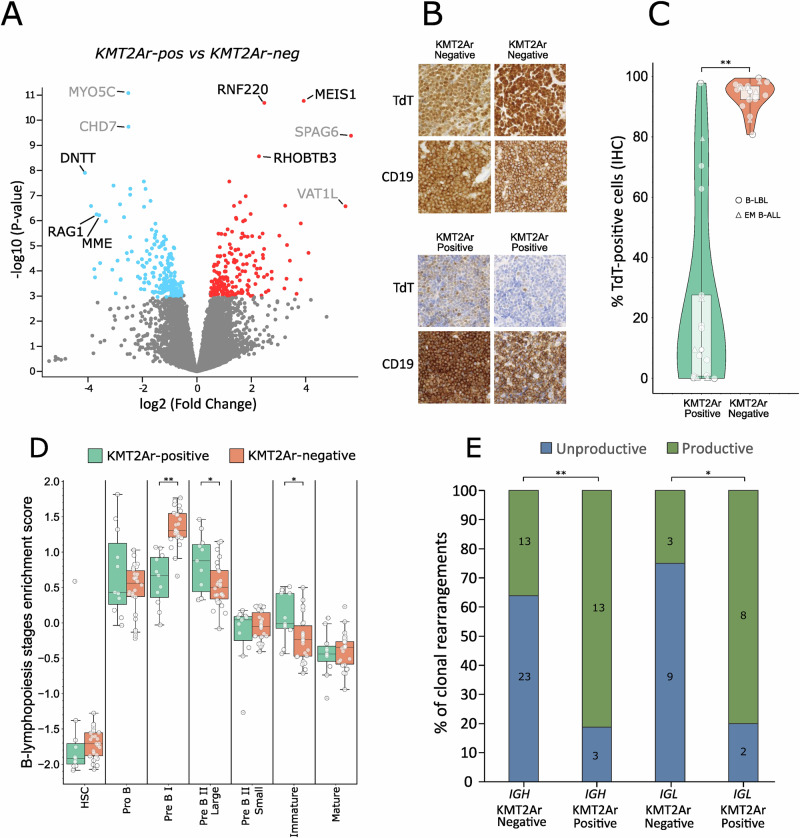
KMT2A rearrangements in B-LBL are characterized by low TdT expression and a more mature stage of cellular differentiation. **A** Volcano plot showing the most significantly differentially expressed genes in the comparison of 11 biopsies from KMT2Ar-positive B-LBL and EM B-ALL samples with 25 biopsies from KMT2Ar-negative B-LBL and EM B-ALL samples. The genes displayed in black are either known KMT2Ar-associated genes (*MEIS1*, *RNF220*, *RHOBTB3*) or known markers of precursor B-cells (*DNTT*, *RAG1*, *MME*). **B** Immunohistochemical analysis of TdT and CD19 expression in two representative KMT2Ar-positive B-LBLs samples and two KMT2Ar-negative B-LBLs samples. The expression of CD19 is shown as a measure of tumor cell content. **C** Image analysis-based quantification of TdT expression in the diagnostic slides of 17 B-LBL and EM B-ALL samples stained for TdT in comparison with *n* = 18 matched B-LBL biopsies belonging to other subtypes [2]. B-LBL samples are labelled with circles and EM B-ALL samples are labelled with triangles. The analysis was performed on representative regions of interest with high tumor-cell content, using the open source software for digital image analysis QuPath [15] and the StarDist segmentation algorithm (arXiv:1806.03535). **D** Analysis of the developmental trajectories of the same samples analysed in (**A**) performed using the ALLCatchR classifier [6]. **E** Analysis of *IGH* and *IGL* rearrangements in 13 KMT2Ar-positive and 24 KMT2Ar-negative B-LBL samples. The analysis was performed on data obtained using the Euroclonality Capture assay. Statistical significance has been assessed using a two-tailed Student’s t-test for panels (**C**, **D**) and a two-tailed Fisher’s Exact test for the data presented in panel (**E**). (*) is used for p < 0.05; (**) for p < 0.005.

RNA-sequencing (RNA-seq) data was available for five KMT2Ar B-LBL from a previous study [2], and we added RNA-seq of additional six B-LBL/EM B-ALL cases defined as KMT2Ar by FISH. RNA-seq data of overall 11 B-LBL/EM B-ALL KMT2Ar specimens was compared to data from 25 patients without KMT2Ar for which molecular subtypes were either previously published [2], or defined based on newly generated data (Table S1). Mutational analysis from the RNA-seq data confirmed an established observation [3], that KMT2Ar samples have overall fewer cancer driver mutations (Figure S1). The unsupervised analysis shown in Figure S2, shows that patients cluster according to the KMT2Ar status and not depending on material type, justifying lumping together B-LBL with EM B-ALL. Among the most significantly up-regulated genes in KMT2Ar tissue samples (Fig. 1A), we identified genes previously found to be up-regulated in KMT2Ar B-ALL or AML (e.g. *MEIS1*, *RNF220* and *RHOBTB3*) [4–6], suggesting that transcriptional changes associated with KMT2Ar in B-LBL/EM B-ALL mirror, at least in part, those observed in acute leukemias. The differential gene expression analysis shown in Fig. 1A also highlights a significant down-regulation of *DNTT* (DNA Nucleotidylexotransferase), *RAG1* (Recombination Activating Gene 1) and *MME* (Membrane Metalloendopeptidase, the gene coding for CD10) in B-LBL/EM B-ALL KMT2Ar samples. Accordingly, gene set enrichment analysis (GSEA) showed a significant enrichment of genes up-regulated in KMT2Ar leukemias among the genes up-regulated in the KMT2Ar-positive tissue biopsies, and enrichment of genes specific to lymphocyte progenitors, among the down-regulated genes (Figure S3).

Both *DNTT* and *RAG1* are genes typically expressed in the early stages of B-cell differentiation during VDJ-recombination. The expression of Terminal Deoxynucleotidyltransferase (TdT), the product of the *DNTT* gene, is indeed used as a diagnostic marker to distinguish precursor B-cell neoplasms from mature B-cell neoplasms [7]. Analysis of TdT expression in representative cases of KMT2Ar-negative and -positive B-LBL biopsies by immunohistochemistry confirmed that TdT expression is strongly reduced in KMT2Ar-positive samples (Fig. 1B). To assess the significance of this observation, we performed image analysis of immunohistochemical stainings from 17 of the KMT2Ar biopsies to quantify at the protein level TdT expression in comparison with a matched control group of B-LBL/EM B-ALL cases belonging to other molecular subtypes (*n* = 18, Table S1). As shown in Fig. 1C, we found that the fraction of TdT-positive cells was significantly lower in the KMT2Ar group, with some patients being negative for TdT expression.

Similar to *DNTT* and *RAG1*, CD10, is also known to be transiently expressed in early stages of B-cell differentiation [8]. Reduced expression of these genes in KMT2Ar B-LBL and EM B-ALL biopsies may therefore suggest that these neoplasms originate either from a very immature, stem-cell like cell, or from a cell that has completed the process of VDJ recombination and is representing a rather mature stage of B-cell development. In order to distinguish between these two possibilities, we analysed the RNA-seq data of our samples using the ALLCatchR classifier [6], that uses enrichment scores derived from seven B-cell differentiation stages to predict the cell of origin of lymphoma/leukemia samples. Interestingly, as shown in Fig. 1D, there was a significant reduction in the Pre BI score and an increase in Pre BII Large and Immature scores in the KMT2Ar biopsies, suggesting that the low expression of *DNTT*, *RAG1* and *MME* reflect a rather mature stage of B-cell differentiation as the cell of origin.

Rearrangements in the immunoglobulin (*IG*) loci are known to proceed following a chronological order [9]. Due to the addition of a random number of nucleotides in the process of segments joining, rearrangements can be productive when the open-read frame required to produce a functional receptor is maintained, or unproductive in the case of a shift in the open-reading frame [10]. Given that only B-cells with a productive rearrangement will proceed differentiation to a mature stage of development, we investigated the frequency of productive *IG* rearrangements comparing KMT2Ar-positive and-negative B-LBL/EM B-ALL biopsies. As shown in Fig. 1E, we found a significantly higher number of productive clonal rearrangements of the *IGH* and *IGL* genes in KMT2Ar-positive B-LBL/EM B-ALL biopsies. Nevertheless, sequence analysis of the productive VDJ rearrangements, showed that these rearrangements were of germline sequence and did not show any sign of somatic hypermutation (data not shown). We conclude that B-LBL/EM B-ALL with KMT2Ar are characterized by a transcriptional profile and *IG* rearrangements suggesting a cell of origin reflecting a mature stage of B-cell differentiation which nevertheless is still compatible with a precursor cell.

**Fig. 2:**
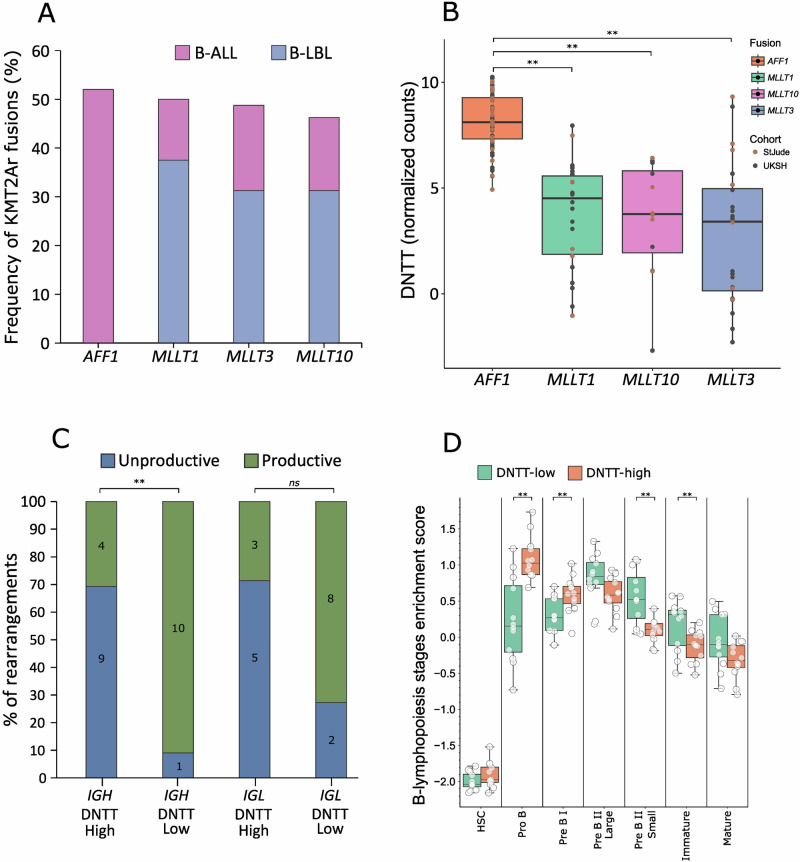
B-ALL subgrouping based on DNTT expression. **A** Comparison of the frequency of *KMT2A* fusion partners in the here described B-LBL KMT2Ar cohort and in KMT2Ar pediatric patients of the StJude and BFM B-ALL cohorts. **B** Analysis of *DNTT* expression in KMT2Ar samples from the StJude and BFM cohorts divided according to the *KMT2A* fusion partner. **C** Analysis of *IGH* and *IGL* rearrangements in 12 KMT2Ar-positive B-ALL samples of the BFM cohort with high DNTT expression and 12 KMT2Ar-positive B-ALL samples with low DNTT expression. The analysis was performed on data obtained using the Euroclonality Capture assay. **D** Analysis of the developmental trajectories of the same samples analysed in (**C**) performed using the ALLCatchR classifier [6]. Statistical significance has been assessed using a two-tailed Student’s t-test for panels (**B**, **D**) and a two-tailed Fisher’s Exact test for the data presented in panel (**C**). (**) for p < 0.005, (*ns*) for not significant.

The *KMT2A* gene is known to be involved in balanced chromosomal aberrations with many different partners. Across all age groups, the most frequent fusion partner for *KMT2A* is *AFF1* [11]. Although not as frequent as in adult cases, in pediatric patients, the *KMT2A::AFF1* fusion accounts for approximately half of all fusions involving *KMT2A*. Therefore, we analysed our B-LBL/EM B-ALL KMT2Ar samples to define the fusion partner for each of them either by RNA-seq data as described previously [2] or by FISH with probes detecting fusions of *KMT2A* with *AFF1*, *MLLT1*, *MLLT3*, and *MLLT10*. In total, we were able to identify a fusion partner in 16 of the 20 KMT2Ar samples. Interestingly, in line with what we published previously on a limited number of samples [1], we failed to detect *KMT2A::AFF1* fusions in our cohort. Fusion partners of *KMT2A* were *MLLT1* (38%), *MLLT3* (31%), and *MLLT10* (31%) (Fig. 2A). One patient had a *PICALM::MLLT10* fusion and was included in our cohort because the case was defined as KMT2Ar by the ALLCatchR classifier.

Recent single and bulk RNA-seq analyses on KMT2Ar in both B-ALL and AML samples have shown the existence of two transcriptional subgroups in the KMT2Ar subtype, one with an expression pattern resembling early/multipotent cells, the other with features of committed/mature cells [12–14]. Having shown that most of the KMT2Ar B-LBL cases express very low TdT and have a strongly biased distribution of *KMT2A* fusion partners, with fusions only involving the *MLLT1*, *MLLT3*, and *MLLT10* genes, we asked what was the distribution of *DNTT* expression in publicly available transcriptomic data of paediatric B-ALL cases. Interestingly, as shown in Fig. 2B, we found that, while almost all *KMT2A::AFF1* fusions had high *DNTT* expression, fusions of *KMT2A* with *MLLT1*, *MLLT3* and *MLLT10* showed a heterogeneous distribution with some samples expressing high *DNTT* and some expressing low *DNTT*. Finally, and in line with published data by Hartmann et al. [14], we found that a set of pediatric B-ALLs expressing low *DNTT* had a transcriptionally more mature phenotype and a higher number of productive *IG* rearrangements (Fig. 2C, D).

In summary, the analysis of a unique cohort of pediatric patients has shown that B-LBL and tissue-derived B-ALL samples of the KMT2Ar subtype, although typically displaying few concomitant driver mutations, are characterized by a mature phenotype and do not display any *KMT2A::AFF1* fusions. These data differ significantly from what has been observed in BM/blood-derived KMT2Ar pediatric B-ALL patients, where approximately 70% show an immature phenotype and approximately 50% carry an *KMT2A::AFF1* fusion [14]. The data here presented may suggest that KMT2Ar of more committed/mature cells is a prerequisite for high tissue dissemination accompanied by low BM infiltration. This hypothesis is supported by the GSEA analysis presented in Figures S3 showing enrichment of genes linked to invasiveness and motility in the KMT2Ar biopsies. Furthermore, given that a high immature/multipotent cellular phenotype has been associated with an adverse outcome and slow decline in MRD under therapy [12, 14], our findings may explain why the KMT2Ar subtype in B-LBL is not associated with unfavourable outcome [1]. Interestingly, as shown in Figure S4, a MYC signature, known to be associated with aggressiveness, was significantly reduced in DNTT-low B-ALL samples. Therefore, our data suggest that tissue-based KMT2Ar cases may represent a biologically distinct, potentially less aggressive subtype, which could influence risk stratification and treatment monitoring.

## Supplementary information

Supplementary Information

## Data Availability

RNA-seq data have been deposited in the European Genome-phenome Archive (accession number EGAD50000002047) and are available upon request.
