# Supplementary material for: KMT2A-rearranged B-lymphoblastic lymphomas are skewed towards a more mature developmental stage
Source: Leukemia. 2026 Mar 23;40(5):1067–71. doi: 10.1038/s41375-026-02943-0 (PMC13148986; doi:10.1038/s41375-026-02943-0)
Supplement: Supplementary file 1 — Supplementary Information [file 41375_2026_2943_MOESM1_ESM.pdf]

Supplementary Information

KMT2A-rearranged B-lymphoblastic lymphomas are skewed towards a more mature developmental stage

Ingram Iaccarino[1,2,3*], Alina M. Hartmann[2,3,4], Fatih Yalcin[1,2,3], Mayukh Mondal[2,5,6], Cecilia Bozzetti[3,4], Nadine Wolgast[2,3,4], Jan L. C. Loeffen[7], Judith M. Boer[7], Monika Brüggemann[2,3,4], Claudia D. Baldus[2,3,4], Gunnar Cario[2,3,8] and Wolfram Klapper[1,2,3*]

[1] Department of Pathology, Hematopathology Section and Lymph Node Registry, University of Kiel, Kiel, Germany

[2] Clinical Research Unit CATCH ALL (KFO 5010) funded by the Deutsche Forschungsgemeinschaft (DFG, German Research Foundation), Kiel, Germany

[3] University Cancer Center Schleswig-Holstein (UCCSH), University Medical Center Schleswig-Holstein, Germany

[4] Medical Department II, Hematology and Oncology, University Hospital Schleswig-Holstein, Kiel, Germany

[5] School of Biology, Indian Institute of Science Education and Research, Thiruvananthapuram, India

[6] Institute of Clinical Molecular Biology, University of Kiel, Kiel, Germany

[7] Princess Máxima Center for Pediatric Oncology, Utrecht, the Netherlands

[8] Department of Pediatrics I, Pediatric Hematology/Oncology, University Medical Center Schleswig-Holstein, Campus Kiel, Kiel, Germany

*To whom correspondence should be addressed: iiaccarino@path.uni-kiel.de; wklapper@path.uni-kiel.de

Description of additional Supplementary Files

- **Supplementary Methods**
- **Supplementary Table S1:** Molecular features of the patient cohort used in this study.
- **Supplementary Table S2:** Enrichments scores for B-cell differentiations stages.
- **Supplementary Table S3:** Genes common between DNTT-low B-ALL and KMT2Ar-positive B-LBL.
- **Supplementary Figure S1:** Mutation analyses**.**
- **Supplementary Figure S2:** Unsupervised analysis of B-LBL samples.
- **Supplementary Figure S3:** GSEA analysis of KMT2Ar B-LBL.
- **Supplementary Figure S4:** GSEA analysis of DNTT-low B-ALL.

Supplementary Materials and Methods

RNA-sequencing

RNA was extracted using the RecoverAll™ Total Nucleic Acid Isolation Kit for FFPE (Thermo Fisher Scientific, CA, USA) and quantified using a Qubit 2.0 Fluorometer (Thermo Fisher Scientific, CA, USA). RNA quality was assessed by capillary electrophoresis (TapeStation, Agilent). Only RNA samples with a DV200 (percentage of fragments of >200 nucleotides) higher than 30% were considered for sequencing. To improve the success rate of RNA-seq from low quality RNAs from FFPE, an exome capture RNA-seq approach was used (1). Total RNA libraries were prepared with the xGen Broad-Range RNA Library Prep Kit (Integrated DNA Technology, USA). To select for sequences of coding genes, libraries were captured in pools of four with the xGen Exome Panel V2 (Integrated DNA Technology, USA) using the xGen Hybridization and Wash Kit (Integrated DNA Technology, USA).
Pre-processing of the data was performed with the nf-core's (2) RNA-seq pipeline (3.11.2) using GRch38 as reference genome and GENCODE version 42. STAR and Salmon were used for genome alignment and transcriptome quantification. RNA-fusions detection was performed using Arriba (3). RSeQC generated a multi-quality control report. Across 36 RNA-seq samples, we observed an average duplication rate of ~52%, a highly consistent GC content (~52% ± 1.2%) and ~122M reads per sample. Overall, QC metrics indicated technically sound and consistent libraries.
The RNA-seq data were analysed on the Galaxy web platform (4) using the limma package with the voom transformation. Raw count data were transformed to log2-counts per million and weighted to account for the mean–variance relationship. This transformation allows the data to approximately meet the assumptions of the linear model used for differential expression analysis.
Normalized gene expression data was used to predict subtypes using the ALLCatchR classifier (5). SNVs analysis was performed from the same samples using the IntegrateALL pipeline (6) starting from the Fastq files. Identified mutations were confirmed at the DNA level using data from the Euroclonality Assay.

Analysis of IG gene rearrangements

IG rearrangement analysis was performed using the EuroClonality-NDC Assay (Univ8 Genomics. Belfast, UK), according to the manufacturer. Briefly, 65 ng of DNA libraries prepared using the KAPA Library Preparation kit (KAPA Biosystems, MA, USA), were pooled and captured with 4 µl of the EuroClonality-NDC Assay, using the KAPA HyperCapture Reagent Kit and the KAPA HyperCapture Bead Kit (KAPA Biosystems, MA, USA). The target-enriched pool was sequenced on an Illumina NextSeq

500/550 system using a 75 bp paired-end strategy performed on a 150-cycle NextSeq 500/550 Mid Output Kit (Illumina, San Diego, CA, USA). Data were analyzed using the ARResT/Interrogate bioinformatic platform (http://arrest.tools/interrogate-latest/).

Image analysis

The analysis of TdT and CD19 expression in diagnostic slides of 35 B-LBL and EM B-ALL biopsies was performed using the open source software for digital image analysis QuPath (7) and the StarDist segmentation algorithm (arXiv:1806.03535). Quantification of CD19 expression was performed in order to select for regions of interest (ROI) with high tumor-cell content. For each slide at least three ROI were used to quantify the % of cells expressing TdT.

Supplementary Tables

Supplementary Table S1: Molecular features of the patient cohort used in this study.

Pt_ID	Age	Sex	Type	Subtype	RNA-Seq	IG Rearr.	Also in Kroeze (8)	%TdT pos	DNTT (counts)	KMT2A BA (FISH)	Fusions (FISH)	Fusions (RNAseq)
**B-LBL030**	1	female	B-LBL	KMT2A	No	yes	yes	6.2	n.a.	Positive	n.a.	n.a.
**B-LBL031**	2	female	B-LBL	ETV6::RUNX1	yes	no	yes	n.a.	9.2	n.a.	n.a.	ETV6::RUNX1
**B-LBL098**	8	female	EM_B-ALL	Low hypodiploid	yes	no	no	n.a.	11.7	n.a.	n.a.	negative
**B-LBL085**	16	male	B-LBL	TCF3::PBX1	No	yes	yes	92.5	10.6	Negative	n.a.	TCF3::PBX1
**B-LBL099**	16	female	EM_B-ALL	iAMP21	yes	no	no	n.a.	10.0	n.a.	n.a.	negative
**B-LBL100**	4	male	EM_B-ALL	Hyperdiploid	yes	no	no	n.a.	11.4	n.a.	n.a.	negative
**B-LBL006**	4	female	B-LBL	KMT2A	yes	yes	yes	n.a.	7.4	n.a.	n.a.	KMT2A::MLLT3
**B-LBL070**	9	female	B-LBL	KMT2A	yes	yes	yes	0.8	8.1	Positive	n.a.	KMT2A::MLLT1
**B-LBL007**	4	male	B-LBL	ETV6::RUNX1	yes	yes	yes	95.7	10.7	n.a.	n.a.	ETV6::RUNX1
**B-LBL051**	7	male	B-LBL	Hyperdiploid	yes	no	yes	n.a.	10.7	n.a.	n.a.	negative
**B-LBL008**	6	female	B-LBL	KMT2A	yes	yes	yes	27.7	6.8	Negative	n.a.	PICALM::MLLT10
**B-LBL009**	5	male	B-LBL	Hyperdiploid	yes	no	yes	n.a.	10.1	n.a.	n.a.	MTO1::JPH3
**B-LBL101**	0	female	EM_B-ALL	KMT2A	yes	yes	no	79.4	10.2	Positive	n.a.	negative
**B-LBL052**	10	female	B-LBL	HeH	yes	yes	yes	94.7	10.6	Negative	n.a.	n.a.
**B-LBL042**	9	male	B-LBL	iAMP21	No	yes	yes	98.2	5.9	Negative	n.a.	n.a.
**B-LBL102**	3	female	B-LBL	KMT2A	No	no	no	70.5	n.a.	Positive	KMT2A::MLLT3	n.a.
**B-LBL011**	6	female	B-LBL	ETV6::RUNX1	yes	yes	yes	86.8	8.8	Negative	n.a.	ETV6::RUNX1
**B-LBL032**	4	female	B-LBL	Hyperdiploid	yes	no	yes	n.a.	9.5	Negative	n.a.	negative
**B-LBL012**	2	male	B-LBL	NUTM1	yes	yes	yes	93.3	10.3	Negative	n.a.	ZNF618::NUTM1
**B-LBL013**	3	female	B-LBL	Other	yes	no	yes	n.a.	8.8	Negative	n.a.	ZNF710::CRTC3
**B-LBL033**	3	female	B-LBL	KMT2A	yes	yes	yes	97.8	9.5	Positive	KMT2A::MLLT1	KMT2A::MLLT1
**B-LBL053**	12	male	B-LBL	Ph-like	yes	yes	yes	90.3	11.7	Negative	n.a.	PAX5::JAK2
**B-LBL071**	9	male	B-LBL	ETV6::RUNX1	yes	no	yes	n.a.	10.0	Negative	n.a.	ETV6::RUNX1
**B-LBL072**	9	female	B-LBL	NUTM1	yes	yes	yes	96.8	11.9	Negative	n.a.	KAT6A::NUTM1

**B-LBL082**	13	male	B-LBL	HeH	yes	yes	yes	92.4	11.4	Negative	n.a.	n.a.
**B-LBL103**	1	female	B-LBL	KMT2A	No	no	no	7.6	n.a.	Positive	KMT2A::MLLT10	n.a.
**B-LBL002**	4	male	B-LBL	NUTM1	No	yes	yes	80.9	10.2	Negative	n.a.	CUX1::NUTM1
**B-LBL034**	4	male	B-LBL	HeH	yes	yes	yes	96.9	11.3	Negative	n.a.	n.a.
**B-LBL015**	4	male	B-LBL	ETV6::RUNX1	yes	no	yes	n.a.	8.8	Negative	n.a.	ETV6::RUNX1
**B-LBL104**	9	female	EM_B-ALL	HeH	No	yes	no	85.4	10.5	n.a.	n.a.	n.a.
**B-LBL105**	12	male	EM_B-ALL	Hyperdiploid	yes	no	no	n.a.	12.3	n.a.	n.a.	negative
**B-LBL066**	10	female	B-LBL	DUX4	No	yes	yes	99.5	10.0	n.a.	n.a.	DUX4::IGH
**B-LBL027**	3	female	B-LBL	PAX5alt	No	yes	yes	97.5	11.0	n.a.	n.a.	n.a.
**B-LBL106**	4	male	EM_B-ALL	HeH	yes	yes	no	98.2	11.0	n.a.	n.a.	negative
**B-LBL016**	1	female	B-LBL	Other	yes	no	yes	n.a.	9.0	n.a.	n.a.	PAX5::ZNF521
**B-LBL107**	16	female	EM_B-ALL	DUX4	yes	no	no	n.a.	10.9	n.a.	n.a.	DUX4::IGH
**B-LBL108**	7	male	EM_B-ALL	ETV6::RUNX1	yes	yes	no	96.2	11.2	n.a.	n.a.	ETV6::RUNX1
**B-LBL109**	6	male	EM_B-ALL	HeH	yes	yes	no	93.9	10.3	n.a.	n.a.	negative
**B-LBL110**	1	female	EM_B-ALL	KMT2A	yes	yes	no	9.6	6.4	n.a.	n.a.	KMT2A::MLLT1
**B-LBL111**	11	male	EM_B-ALL	Hyperdiploid	yes	no	no	n.a.	11.6	n.a.	n.a.	negative
**B-LBL112**	1	female	EM_B-ALL	KMT2A	yes	no	no	0.3	2.1	Positive	n.a.	KMT2A::MLLT3
**B-LBL113**	1	male	B-LBL	KMT2A	No	no	no	n.a.	n.a.	Positive	KMT2A::MLLT1	n.a.
**B-LBL114**	4	male	B-LBL	KMT2A	yes	no	no	1.0	1.8	Positive	n.a.	KMT2A::MLLT10
**B-LBL115**	2	male	B-LBL	KMT2A	yes	yes	no	17.6	5.2	n.a.	n.a.	KMT2A::MLLT3
**B-LBL116**	7	male	B-LBL	KMT2A	yes	yes	no	62.9	7.5	Positive	n.a.	KMT2A::MLLT1
**B-LBL117**	15	female	B-LBL	KMT2A	yes	yes	no	16.6	5.5	Positive	n.a.	KMT2A::MLLT1
**B-LBL118**	1	female	B-LBL	KMT2A	No	no	no	0.3	n.a.	Positive	n.a.	n.a.
**B-LBL119**	5	male	EM_B-ALL	ETV6::RUNX1	No	no	no	96.5	n.a.	n.a.	ETV6::RUNX1	n.a.
**B-LBL120**	2	male	EM_B-ALL	KMT2A	No	yes	no	0.1	n.a.	Positive	n.a.	n.a.
**B-LBL121**	2	female	EM_B-ALL	KMT2A	No	yes	no	0.1	n.a.	Positive	KMT2A::MLLT10	n.a.
**B-LBL122**	0	male	EM_B-ALL	KMT2A	No	yes	no	26.4	n.a.	Positive	KMT2A::MLLT3	n.a.
**B-LBL037**	1	female	B-LBL	KMT2A	No	no	yes	n.a.	3.3	n.a.	n.a.	KMT2A::MLLT10

Supplementary Table S2: Enrichments scores for B-cell differentiations stages.

Pt_ID	Age	Type	Subtype	HSC	Pro B	Pre BI	Pre BII Large	Pre BII Small	Immature	Mature
**B-LBL031**	2	B-LBL	ETV6::RUNX1	-1.812	0.617	1.386	0.323	0.210	-0.385	-0.340
**B-LBL098**	8	EM_B-ALL	Low hypodiploid	-1.641	-0.108	1.203	1.151	-0.185	0.237	-0.658
**B-LBL099**	16	EM_B-ALL	iAMP21	-1.711	0.461	1.582	0.136	0.240	-0.477	-0.231
**B-LBL100**	4	EM_B-ALL	Hyperdiploid	-1.512	0.534	1.670	0.404	-0.185	-0.573	-0.338
**B-LBL006**	4	B-LBL	KMT2A	-1.917	-0.036	1.069	0.877	-0.017	0.464	-0.441
**B-LBL070**	9	B-LBL	KMT2A	-1.970	0.927	0.895	0.539	0.095	-0.097	-0.388
**B-LBL007**	4	B-LBL	ETV6::RUNX1	-1.468	0.053	1.682	0.598	0.231	-0.547	-0.549
**B-LBL051**	7	B-LBL	Hyperdiploid	-1.434	0.388	1.766	0.294	-0.024	-0.712	-0.278
**B-LBL008**	6	B-LBL	KMT2A	-1.380	1.816	0.430	0.345	-0.469	-0.377	-0.365
**B-LBL009**	5	B-LBL	Hyperdiploid	-1.841	0.438	1.250	0.743	0.079	-0.141	-0.527
**B-LBL101**	0	EM_B-ALL	KMT2A	-2.084	0.796	0.952	0.325	0.082	-0.057	-0.014
**B-LBL052**	10	B-LBL	HeH	-1.870	1.029	1.104	-0.084	-0.408	0.000	0.228
**B-LBL011**	6	B-LBL	ETV6::RUNX1	-1.437	-0.220	1.655	0.727	0.227	-0.237	-0.716
**B-LBL032**	4	B-LBL	Hyperdiploid	-1.920	0.645	1.296	0.391	-0.068	-0.149	-0.195
**B-LBL012**	2	B-LBL	NUTM1	-2.020	0.112	0.661	1.138	0.053	0.323	-0.266
**B-LBL013**	3	B-LBL	Other	-1.735	-0.132	1.291	0.790	-0.059	0.500	-0.654
**B-LBL033**	3	B-LBL	KMT2A	-1.758	0.346	1.056	1.129	-0.144	-0.013	-0.616
**B-LBL053**	12	B-LBL	Ph-like	-1.607	0.831	1.397	0.519	-0.302	-0.238	-0.600
**B-LBL071**	9	B-LBL	ETV6::RUNX1	-1.611	0.631	1.525	0.242	0.236	-0.661	-0.361
**B-LBL072**	9	B-LBL	NUTM1	-1.710	0.419	1.210	0.936	0.165	-0.427	-0.594
**B-LBL082**	13	B-LBL	HeH	-1.910	0.778	1.222	0.410	-0.199	-0.157	-0.143
**B-LBL034**	4	B-LBL	HeH	-1.554	0.587	1.549	0.576	-0.184	-0.464	-0.510
**B-LBL015**	4	B-LBL	ETV6::RUNX1	-1.277	-0.178	1.530	1.039	-0.003	-0.170	-0.943
**B-LBL105**	12	EM_B-ALL	Hyperdiploid	-1.533	0.407	1.723	0.383	-0.155	-0.472	-0.352
**B-LBL106**	4	EM_B-ALL	HeH	-1.650	0.795	1.466	0.286	-0.044	-0.586	-0.267
**B-LBL016**	1	B-LBL	Other	-2.071	0.688	0.911	0.546	0.019	0.183	-0.274
**B-LBL107**	16	EM_B-ALL	DUX4	-1.560	0.751	1.262	0.852	-0.338	-0.257	-0.710
**B-LBL108**	7	EM_B-ALL	ETV6::RUNX1	-1.946	0.369	1.312	0.486	0.187	-0.196	-0.213
**B-LBL109**	6	EM_B-ALL	HeH	-2.004	0.827	1.063	0.165	-0.296	0.230	0.015
**B-LBL110**	1	EM_B-ALL	KMT2A	-1.659	0.072	0.724	1.463	0.174	-0.037	-0.738
**B-LBL111**	11	EM_B-ALL	Hyperdiploid	-1.761	0.954	1.186	0.545	-0.299	-0.293	-0.331
**B-LBL112**	1	EM_B-ALL	KMT2A	-1.899	0.431	0.670	1.084	-0.350	0.511	-0.447
**B-LBL114**	4	B-LBL	KMT2A	-2.025	1.321	0.148	0.342	0.076	0.071	0.067
**B-LBL115**	2	B-LBL	KMT2A	-1.961	0.178	0.292	1.334	0.042	0.412	-0.297
**B-LBL116**	7	B-LBL	KMT2A	-2.023	0.416	0.509	1.033	0.119	0.416	-0.471
**B-LBL117**	15	B-LBL	KMT2A	0.587	1.472	-0.029	0.742	-1.269	-0.437	-1.066
**B-LBL037**	1	B-LBL	KMT2A	-2.044	-0.023	0.502	1.000	0.654	0.218	-0.306

Enrichments scores for B-cell differentiations stages obtained using the ALLCatchR classifier as in Beder et al (5)

Supplementary Table S3: Genes common between DNTT-low B-ALL and KMT2Ar-positive B-LBL.

Gene.name	DNTT-low_vs_High_B-ALL			KMT2Ar-pos_vs_Neg_B-LBL		
	logFC	P.Value	adj.P.Val	logFC	P.Value	adj.P.Val
COL19A1	6.13	0.0081362	0.1913158	3.82	1.28E-06	0.0006949
SPAG6	4.91	0.0014972	0.1065566	5.66	4.14E-10	1.44E-06
CFAP46	3.73	0.0000134	0.0257406	2.15	0.0003325	0.0251266
TNNT3	3.39	0.0000434	0.0345402	1.27	0.0006551	0.0369689
FGF9	3.06	0.0010959	0.0954995	2.75	0.0001068	0.0122986
FAM110C	2.83	0.0010726	0.0954995	4.10	1.92E-05	0.0042142
ADD2	2.74	0.0002378	0.0608463	2.18	0.0001244	0.0136849
TRIB2	2.56	0.0003609	0.0706633	1.70	1.06E-06	0.0005966
CCDC141	2.32	0.005187	0.1626642	2.06	1.85E-05	0.0041655
DTX4	1.90	0.0022775	0.121486	1.80	1.06E-07	0.0001314
DNAJC1	1.38	0.0027057	0.1279268	1.98	5.41E-07	0.0004064
CACFD1	1.36	0.0011454	0.0954995	0.90	0.0001887	0.0181215
NOL4L	1.25	0.0029051	0.1292973	1.00	0.0002119	0.019161
SLC2A11	0.99	0.0049457	0.1598871	0.76	0.0005995	0.035201
SHANK1	0.83	0.0057031	0.1682524	2.05	0.0009798	0.0459177
RASGRP1	0.82	0.0081462	0.1913158	0.87	0.0001843	0.0179947
IFT74	-0.69	0.008925	0.1985406	-0.94	2.72E-05	0.0050512
IGF1R	-1.10	0.0049762	0.1598871	-1.17	0.0002287	0.019697
CCND2	-1.15	0.0080655	0.1909852	-1.93	0.0005471	0.0336141
MLC1	-1.21	0.002139	0.1181555	1.97	0.0009828	0.0459177
CPNE2	-1.24	0.0014443	0.1065566	-1.22	0.0002231	0.0195801
SCARF1	-1.33	0.0007916	0.0846557	-1.34	0.0001501	0.0158137
KCTD3	-1.37	0.000329	0.0693083	-1.01	0.0002524	0.0208871
CHD7	-1.38	0.0079368	0.1894613	-2.51	1.82E-10	7.93E-07
SAV1	-1.50	0.002387	0.1228876	-1.38	1.13E-05	0.0030515
MAGI3	-1.64	0.0048323	0.1595213	-1.14	5.57E-06	0.0018267
GRB10	-1.67	0.0024079	0.1228876	-1.52	1.32E-06	0.0006949
B4GALT6	-1.94	0.0015264	0.1065566	-1.58	0.0007352	0.0391568
EPHB4	-2.02	0.0001032	0.0450206	-0.95	0.0002289	0.019697
MARVELD1	-2.04	0.0026372	0.1259368	-1.20	0.000201	0.0186777
ERG	-2.10	0.0072041	0.1862527	-2.46	4.46E-06	0.0015833
SCML2	-2.29	0.0000193	0.0276639	-2.68	7.25E-07	0.0004848
NPR1	-3.34	0.0026476	0.1260105	-2.16	4.55E-05	0.007084
RAG1	-3.50	0.0000162	0.0257406	-3.69	5.61E-07	0.0004064
ADPRHL1	-4.25	0.0001229	0.0470532	-3.07	3.99E-08	6.94E-05
DNTT	-6.72	5.72E-09	0.0000819	-4.11	1.25E-08	3.10E-05

Fold Change (logFC) and significance (p.Val and adj.p.Val) of genes found differentially expressed in DNTT-low pediatric B-ALL (as in Figure 2D) and in KMT2Ar-positive B-LBL/EM B-ALL samples (as in Figure 1A and D). Although not always reaching a significant adjusted p.Val as single genes, there was a significant enrichment of KMT2Ar-pos_vs_Neg_B-LBL genes in both B-ALL_DNTT-low upregulated (NES=2.14, FDR=0.0) and downregulated (NES=-1.86, FDR= 0.00635) genes.

Supplementary Figures

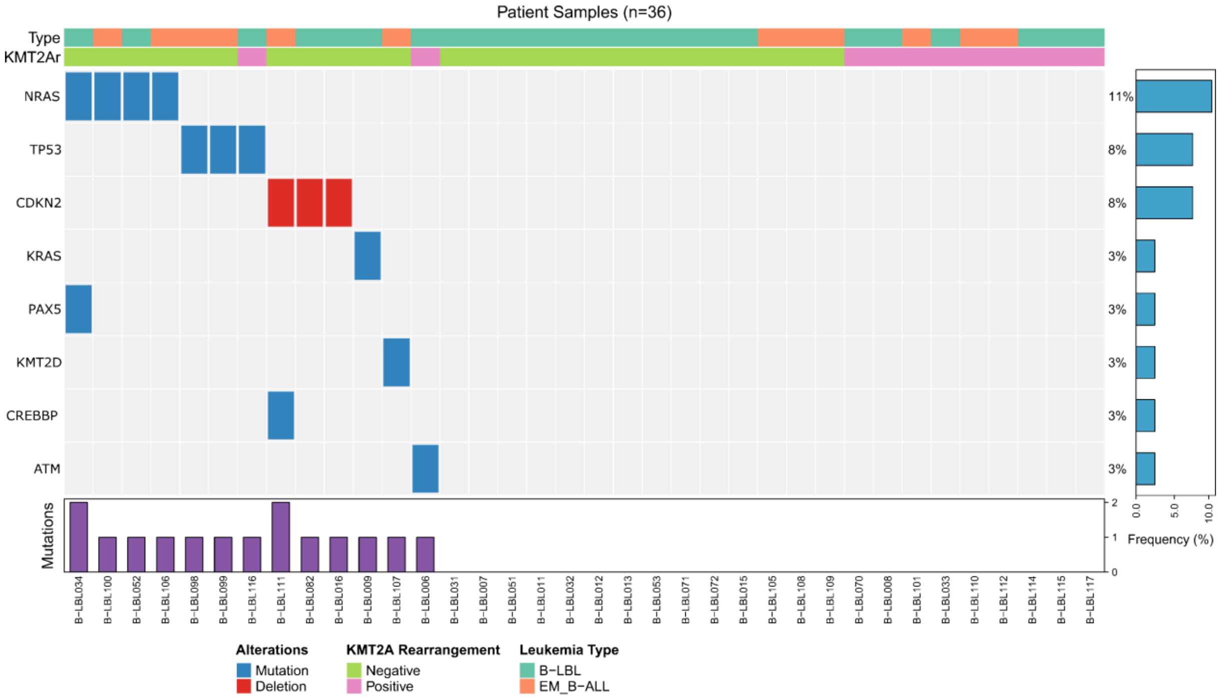

Supplementary Figure S1: Genetics of B-LBL/EM B-ALL KMT2Ar positive and negative samples. The RNA-seq data of the 11 B-LBL/EM B-ALL KMT2Ar-positive and 25 B-LBL/EM B-ALL KMT2Ar-negative samples were analysed using the IntegrateALL pipeline (6) to identify hot spot mutations in a set of leukemia-relevant genes. Mutations were confirmed at the DNA level using data from the EuroClonality assay. CDKN2A/B focal deletions were also identified with the EuroClonality assay. The figure represents an oncoplot with the mutation frequency of the genes found frequently mutated in our samples.

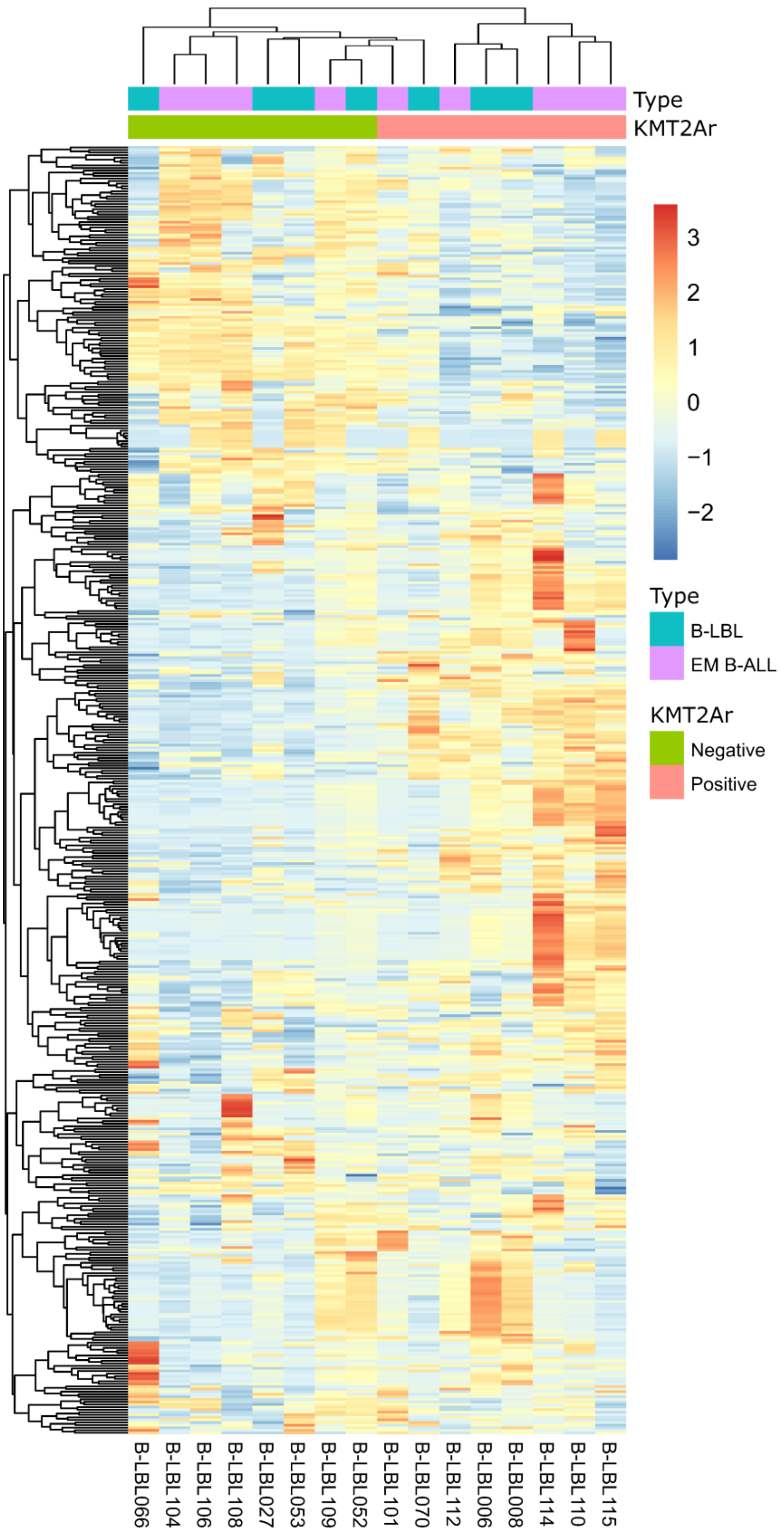

Supplementary Figure S2: Unsupervised analysis of KMT2Ar-positive and negative B-LBL/EM B-ALL samples. The image shows an heatmap of an unsupervised clustering analysis of the top 500 variable genes in a subgroup of 8 KMT2Ar-positive and 8 KMT2Ar-negative B-LBL/EM B-ALL samples.

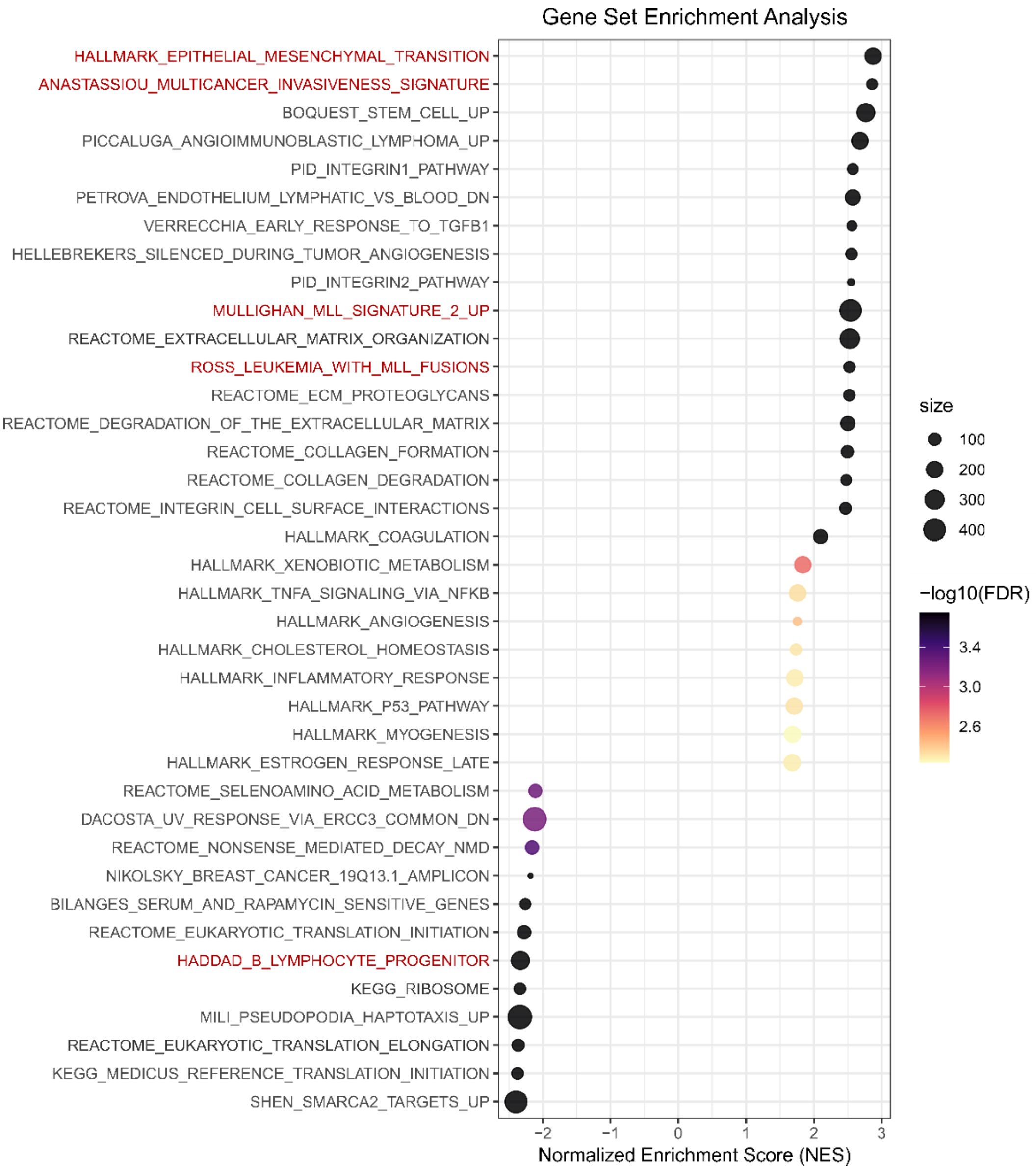

Supplementary Figure S3: Gene set enrichment analyses. Genes expression data from KMT2Ar-positive and KMT2Ar-negative B-LBL and EM B-ALL biopsies (as in Figure 1) was analysed using the GSEA software (version 4.4.0). The results of pathways enriched either in KMT2Ar-positive samples (with a positive Normalized Enrichment Score) or in KMT2Ar-negative samples (with a negative Normalized Enrichment Score) are shown in a bubble plot where the bubble size represent the gene set size and the bubble colour the FDR statistical significance (transformed as -log10). The figure shows that among the up-regulated genes in KMT2Ar-positive biopsies there is a significant enrichment in genes found upregulated in KMT2Ar leukemias (Mullighan_MLL_signature_2_UP, Ross_leukemia_with_MLL_fusions), and in genes associated to degradation of the extracellular matrix and multicancer invasiveness (Epithelial_mesenchimal_transition, Multicancer_invasiveness, Extracellular_matrix_organization). The figure also shows that among the genes down-regulated in KMT2Ar-positive biopsies there is a significant enrichment of genes found associated to B-lymphocyte progenitors (Haddad_B_lymphocyte_progenitor).

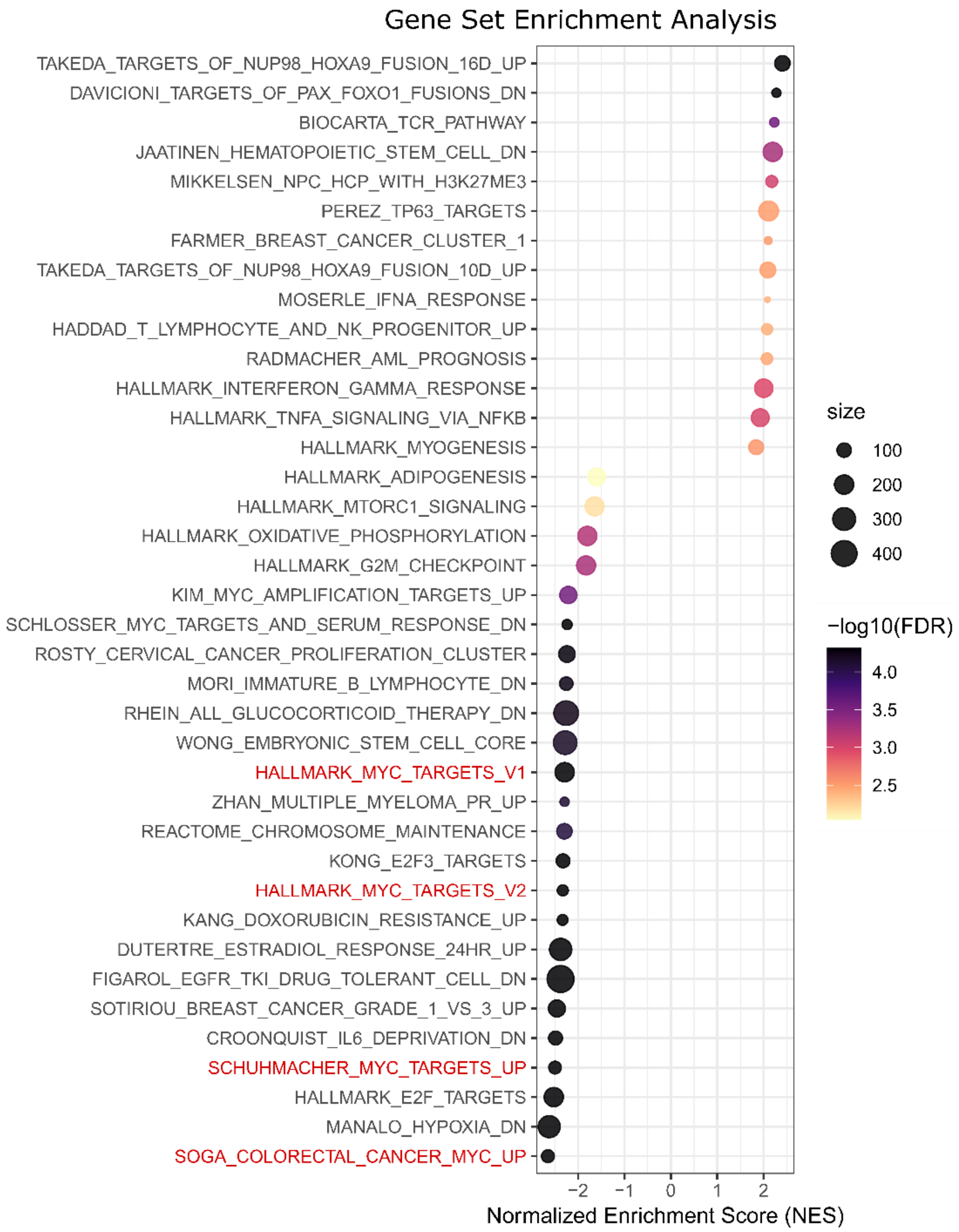

Supplementary Figure S4: Gene set enrichment analyses. Genes expression data from pediatric B-ALL cases divided in DNTT-high and DNTT-low (as in Figure 2) was analysed using the GSEA software (version 4.4.0). The results of pathways enriched either in DNTT-low samples (with a positive Normalized Enrichment Score) or in DNTT-high samples (with a negative Normalized Enrichment Score) are shown in a bubble plot where the bubble size represent the gene set size and the bubble colour the FDR statistical significance (transformed as -log10). The figure shows that among the genes down-regulated in DNTT-low biopsies there is a significant enrichment of several MYC target genes.

Supplementary References

1. Cieslik M, Chugh R, Wu YM, Wu M, Brennan C, Lonigro R, et al. The use of exome capture RNA-seq for highly degraded RNA with application to clinical cancer sequencing. Genome Res. 2015;25(9):1372-81.
2. Ewels PA, Peltzer A, Fillinger S, Patel H, Alneberg J, Wilm A, et al. The nf-core framework for community-curated bioinformatics pipelines. Nat Biotechnol. 2020;38(3):276-8.
3. Uhrig S, Ellermann J, Walther T, Burkhardt P, Frohlich M, Hutter B, et al. Accurate and efficient detection of gene fusions from RNA sequencing data. Genome Res. 2021;31(3):448-60.
4. Galaxy C. The Galaxy platform for accessible, reproducible, and collaborative data analyses: 2024 update. Nucleic Acids Res. 2024;52(W1):W83-W94.
5. Beder T, Hansen BT, Hartmann AM, Zimmermann J, Amelunxen E, Wolgast N, et al. The Gene Expression Classifier ALLCatchR Identifies B-cell Precursor ALL Subtypes and Underlying Developmental Trajectories Across Age. Hemasphere. 2023;7(9):e939.
6. Wolgast N, Beder T, Mondal M, Walter W, Hutter S, Bendig S, et al. IntegrateALL: an end-to-end RNA-seq analysis pipeline for multilevel data extraction and interpretable subtype classification in B-precursor ALL. bioRxiv. 2025.
7. Bankhead P, Loughrey MB, Fernandez JA, Dombrowski Y, McArt DG, Dunne PD, et al. QuPath: Open source software for digital pathology image analysis. Sci Rep. 2017;7(1):16878.
8. Kroeze E, Iaccarino I, Kleisman MM, Mondal M, Beder T, Khouja M, et al. Mutational and transcriptional landscape of pediatric B-cell precursor lymphoblastic lymphoma. Blood. 2024;144(1):74-83.
